# The Beta3 Adrenergic Receptor in Healthy and Pathological Cardiovascular Tissues

**DOI:** 10.3390/cells9122584

**Published:** 2020-12-02

**Authors:** Lauriane Y. M. Michel, Charlotte Farah, Jean-Luc Balligand

**Affiliations:** 1 Pole of Pharmacology and Therapeutics (FATH), Institut de Recherche Experimentale et Clinique (IREC), Université Catholique de Louvain, B1.57.04, 57 Avenue Hippocrate, 1200 Brussels, Belgium; lauriane.michel@uclouvain.be (L.Y.M.M.); charlotte.farah@uclouvain.be (C.F.); 2 Department of Medicine, Cliniques Universitaires Saint-Luc, Université Catholique de Louvain, 10 Avenue Hippocrate, 1200 Brussels, Belgium

**Keywords:** adrenergic receptor, beta, catecholamines, cardiac physiology, myocardial remodeling, metabolism, beige adipocyte, heart failure

## Abstract

The third isotype of beta-adrenoreceptors (β3-AR) has recently come (back) into focus after the observation of its expression in white and beige human adipocytes and its implication in metabolic regulation. This coincides with the recent development and marketing of agonists at the human receptor with superior specificity. Twenty years ago, however, we and others described the expression of β3-AR in human myocardium and its regulation of contractility and cardiac remodeling. Subsequent work from many laboratories has since expanded the characterization of β3-AR involvement in many aspects of cardiovascular physio(patho)logy, justifying the present effort to update current paradigms under the light of the most recent evidence.

## 1. Introduction

Beta3-adrenergic receptors (β3-AR) have traditionally been considered as metabolic receptors in the adipose tissue. After a long period of relative disinterest due to disappointing performance of early agonist drugs, this is now being actively studied again, with new exciting findings in human white or beige adipocytes. In the meantime, the demonstration of β3-AR expression in cardiac myocytes and endothelial cells sparked international efforts from many research groups to decipher the role of this receptor in cardiovascular physiology and pathology. The present review recapitulates current knowledge on the structure, coupling and function of β3-AR in these tissues, with a view to evaluate translational applications in human disease based on the availability of more selective agonists.

## 2. β3-AR Structure

The third isoform of human β-AR was identified by cloning in 1989 [1]. Initially studied in the adipose tissue for its metabolic role (in lipolysis and thermogenesis) and for its regulation of smooth muscle relaxation in the gastroinstestinal tract and urinary bladder, it is now recognized to also regulate the cardiovascular system through its expression in vascular endothelial cells [2,3] and both atrial and ventricular cardiac myocytes [4]. The β3-AR belongs to the G protein-coupled receptors (GPCRs) family, sharing their typical structure with 7 transmembrane domains (3 intra- and 3 extra- loops) with a glycosylated N-terminal extracellular domain and a C-terminal intracellular domain. Depending on the subunit of G protein that it couples with, agonist activation of the receptor will stimulate adenylyl cyclase (AC)/cAMP production (G-alpha-s protein; Gαs) or inhibit it (G-alpha-i protein; Gαi) to the benefit of cGMP production signaling (discussed in part 2, below). β3-AR shares approximatively 50–40% of amino-acid sequence homology with β1 and β2-AR, respectively, with main divergences located in the third intracellular loop and C-terminal tail. The latter contains a S-palmitoylation canonical site on Cys^361/363^, shared by the all β-ARs, involved in G-protein coupling and AC activation in β1/β2AR. A recent study further observed that Cys^361/363^ is involved in β3 receptor-effector coupling (readout by ligand-induced cAMP production) but also in β3-AR abundance and stability, and described new specific human β3-AR S-palmitoylated sites on Cys^153^ and Cys^292^, within the second and third intracellular loops, respectively, which may also regulate membrane receptor abundance (assessed with FLAG-β3-AR immunostaining) [5]. Particularly, based on the lack of serine and threonine residues sequences targeted by GPCR kinases (GRKs) and PKA phosphorylation on the third intracellular loop and C-terminal tail present in β1/β2AR, β3-ARs have been assumed to be resistant to agonist-induced desensitization. Indeed, in response to sustained catecholamine stimulation, G-protein binding to β1/β2AR could be inhibited through the recruitment of the β-arrestin promoted by GRK-dependent phosphorylation (GRK2 in cardiac myocytes) or through the conformational modification of the receptor induced by PKA phosphorylation. The divergence of β3-AR from this classical paradigm was initially demonstrated through the use of chimeric β2/β3-AR, in which the third cytoplasmic loop and the C-terminal tail were exchanged with those of the β2-AR in Chinese hamster fibroblasts (CHW) and murine Ltk- cells (L cells) [6,7]. Several studies, however, reconsidered the potential of the β3-AR to be desensitized [8]. Despite a lot of discrepancy owing to the species, tissue/cell types and methodology applied—especially regarding the pharmacology of β3-AR stimulation and duration of agonist exposure— some studies [9,10], but not others [6,11], reported agonist-induced β3-AR desensitization. Of note is that most of these studies were performed on rodent adipocytes or CHW and HEK293 cells, and used cAMP production as readout (without considering phosphodiesterases (PDE) and AC activities sensitive to IBMX and forskolin, respectively). A more recent study on neonatal rat cardiomyocytes reported that 30 min pre-treatment of cardiomyocytes with 1 μM BRL37344 (a β3-AR specific agonist) diminishes the subsequent cAMP synthesis in response to 10 μM BRL37344. The authors attribute this short-term β3-AR desensitization to be mediated by the regulator of G protein signaling (RGS) homologous domain of GRK2, which regulates the GTP hydrolysis rate (GAP activity) of the G-protein, but not to the kinase activity of the enzyme [12]. However, apart from the fact that such high concentrations of BRL37344 are known to exert off-target effects on β1-ARs, the β3-ARs in human cardiac myocytes are known to be predominantly coupled to Gαi/cGMP signaling, but not Gαs/cAMP signaling [13,14] (see part 2 below). Nevertheless, β-ARs desensitization can manifest itself at a functional level (cyclic nucleotides synthesis), but also at the level of protein expression through receptor downregulation or internalisation from plasma membrane to the cytosol. Unlike β1/β2AR, which are known to be downregulated and desensitized under sustained catecholamine stimulation, β3-AR expression was shown to be upregulated in the myocardium of heart failure (HF) patients [15], but also in animals models of HF [16,17], supporting less propensity for desensitization (see part 3 below).

## 3. β3-AR Expression and Function in the Healthy Heart

### 3.1. β3-AR Expression

Previous reports on expression and function of β3-AR, especially in cardiac tissue, have been controversial owing to the dubious specificity of radioligands and antibodies for protein immunohistological detection (versus β1/β2AR isoforms), and limitations regarding affinity and potency for selective agonists to dissect specific cellular signaling [18]. Moreover interspecies variations in terms of protein expression levels and splice variants coupled to Gαs or Gαi proteins add to the complexity in interpretation [19,20]. With this in mind, in this review, we restrict our focus to the regulation and signaling by the human cardiac whenever possible.

**Figure 1:**
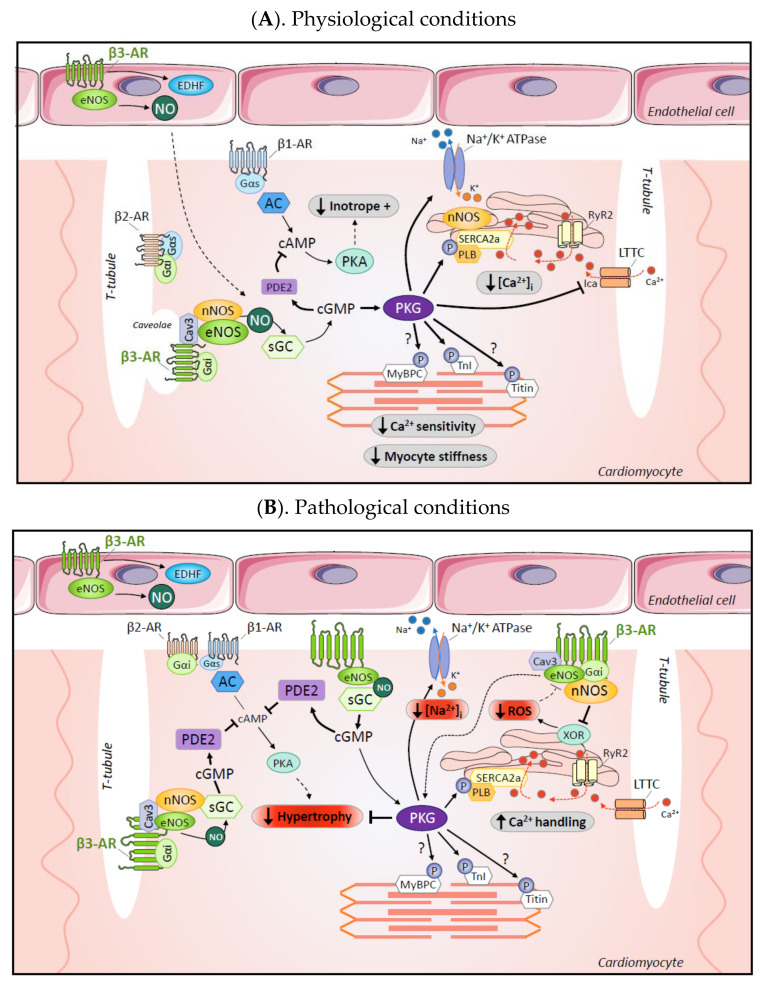
Coupling to cardiac intracellular signaling by the β3-AR in physiological and pathological conditions. (**A**). In physiological conditions, β3-AR are mainly localized in T-tubular membranes where they couple to both eNOS and nNOS; NO production results in the production of a localized pool of cGMP by the soluble Guanylyl Cyclase (sGC); cGMP in turn activates a subset of phosphodiesterases (PDE), including cGMP-activated cAMP PDE or PDE2 that contributes to attenuation of the β1-AR/cAMP-mediated regulation of contractility; cGMP also activates Protein Kinase G (PKG) with downstream phosphorylation of a number of targets modulating contractility—i.e., (i). troponin I (TnI); myosin binding protein C (MyBC) and titin—resulting in decreased myofilament calcium (Ca^2+^) sensitivity and myocyte stiffness (although the connection of β3-AR to the last two is hypothetical at this stage); (ii). phospholamban (PLB) resulting in increased Ca^2+^ re-uptake from cytosol; (iii). beta1 subunit of Na^+^-K^+^ ATPase, promoting its extrusion of Na^+^; in addition, in some mammalian species, PKG decreases Ca^2+^ entry through the L-type Ca^2+^ channel (LTCC), further decreasing cytosolic Ca^2+^. The resulting effect would be a decrease in the inotropic influence of β1/β2-AR activation. In addition, β3-AR expressed in coronary microvascular endothelium produces NO (and EDHF) to increase myocardial perfusion and reinforce the effect of myocyte β3-AR through paracrine effects. (**B**). In pathological conditions, β1AR and β2AR are downregulated and/or desensitized, and migrate out of T-tubules to the “crest”, or peripheral plasma membrane; β3-AR also undergoes a similar translocation, albeit incomplete, and importantly, β3-AR are upregulated and less prone to homologous desensitization, thereby maintaining their intracellular signaling. As in (**A**), signaling through NOS, sGC, cGMP and PDE2 (itself upregulated in the diseased heart) attenuates the residual influence of β1/β2-AR on contractility and remodeling through cAMP/PKA; just as with beta-blockers, in the short term, this β3-AR signaling may decrease inotropy (perhaps justifying the future use of β3-AR antagonists in acute heart failure); but in the long term, β3-AR activation will protect from deleterious effects of β1-AR overstimulation, thereby preventing adverse remodeling, including hypertrophy. In addition, cGMP/PKG signaling will improve Ca^2+^ handling and myocyte relaxation through the same targets as in (**A**). In particular, protection of the beta1 subunit of Na^+^-K^+^ ATPase from oxidation will improve Na^+^ extrusion and reduce deleterious Na^+^ overload. Coupling of β3-AR to nNOS (upregulated in hypertrophic myocardium) will exert additional antioxidant effects through inhibition of Xanthine Oxidoreductase (XOR), resulting in protection of residual eNOS from oxidative uncoupling.

The human β3-AR was first identified in human cardiac biopsies in 1996 [4]. Under physiological conditions, β3-AR are expressed at low levels in myocardial tissue compared to the more abundant β1 and β2-AR, with a representation at approximately 3% versus 80% and 17%, respectively [21]. However, the proportion is altered during disease, as β3-AR are upregulated in failing hearts [15,16,17]. Subsequently, β3-AR was shown to signal through eNOS/NO/cGMP pathway in human ventricle, resulting in attenuation of cardiac contractility ex vivo [13]. Using a transgenic mouse model expressing the human β3-AR specifically in cardiac myocytes together with a FRET-based cGMP biosensor, our group further showed that β3-AR co-localized with caveolin-3 and eNOS in caveolae-enriched rafts, and directly coupled to sGC/cGMP signaling [22] (Figure 1A). More recently, such co-localization was confirmed in experiments combining a similar cGMP biosensor with scanning ion conductance microscopy (SICM) that further identified functional β3-AR to be confined to the T-tubules in healthy rat cardiac myocytes [23].

### 3.2. Inotropic Effect of β3-AR Stimulation

Functionally, β3-AR were initially shown to have inotropic effects antipathetic to β1/β2AR by decreasing cardiac myocyte contractility through eNOS/cGMP signaling in human myocardial biopsies [13], as confirmed in cardiac tissue from the transgenic mouse model with cardiac-specific expression of the human β3-AR [14]; a similar conclusion was drawn from observations of the converse effect in myocytes from β3-AR^−/−^ mice [24]. Another transgenic β3-AR-expressing mouse model yielded opposite effects (i.e., increased contractility) involving the Gαs-mediated pathway, but at vastly higher receptor expression levels, raising questions about promiscuous coupling at supraphysiological receptor abundance [25]. Conversely, the model used by us was shown to express levels of β3-AR protein that matched the abundance observed in human biopsies [26].

Such discrepancies point out that precautions should be taken for the comparison and interpretation of results obtained with acute β3-AR stimulation of human tissues, depending on (i) the origin of the tissue sample (ventricle vs. atrium) and its physio-pathological phenotype (from healthy vs. injured to failing hearts), (ii) the pharmacology applied and (iii) the experimental conditions (e.g., 22 vs. 37 °C).

In human endomyocardial biopsies from the right interventricular septum of cardiac transplanted patients (i.e., non-failing hearts), Gauthier et al. [4] demonstrated that acute β3-AR stimulation with the preferential agonist, BRL37344 induces a dose-dependent negative inotropic effect starting at 0.1 nM with a maximal response obtained at 1 µM. The specificity of β3-AR signaling was confirmed in the presence of β1 and β2-AR antagonists (1 µM metroprolol and 10 µM nadolol) which did not alter the effect, while bupranolol (1 µM; β-AR non-specific antagonist) significantly attenuated the negative tension response of human endomyocardial biopsies. Accordingly, isoprenaline stimulation (0.7 to 10 µM) in the presence of the β1/β2-AR antagonist, nadolol (10 µM), produced a similar attenuation of contractility attributed to β3-AR signaling. Moniotte et al. [15] latter confirmed these results in non-denervated non-failing hearts samples. In contrast, Skeberdis’group [27] did not observe any effect of β3-AR stimulation on contraction force of human left ventricular trabeculae stimulated with the β3-AR agonist CGP12177 (10 µM) in the presence of nadolol (200 nM; i.e., much less than above). However, note in this work that (i) the trabeculae samples were obtained from patients undergoing cardiac surgery for congenital defects, valve replacement or coronary artery bypass graft, involving pathological phenotypes (vs. healthy samples) and (ii) CGP12177 has lower affinity for the human β3-AR compared to BRL37344 or CL316243, with maximal effect observed at 100 µM [4]. On the contrary, the authors observed that β3-AR activation increased the L-type Ca^2+^ channel current (Ica) in isolated human cardiac ventricular myocytes (at odds with the absence of any effect on contractility). Contractile and electrophysiological effects of β3-AR may also differ in atrial myocytes. Although few data are available on this specific tissue, β3-AR stimulation was reported by Skeberdis et al. [28] to slightly increase human right atrial myocyte contractility (10–20% of peak stimulation) by stimulating the L-type Ca^2+^ channel current, through cAMP/PKA signaling [28]. These contractile observations were obtained on atrial trabeculae (from injured hearts) with CGP12177 (1 µM), SR58611 (100 nM) or BRL37344 (1 µM), again in the presence of only 200 nM of nadolol. However, others [29] later refuted this conclusion, based on their observations in different experimental settings closer to physiological conditions. Indeed, in their hands, β3-AR activated human atrial Ica in experiments performed at room temperature (19–25 °C), as in Skeberdis et al. [28], but this effect was lost at 37 °C. No effect of SR58611 or BRL37344 on atrial contractility was observed at 24 °C, while at 37 °C, BRL3734 (but not SR58611) increased contractility in the presence of PDE inhibition (incubation with IBMX; 10 µM), an effect abrogated by β1/β2-AR antagonists, but not by the β3-AR antagonist L-748,337. Similarly, the same L-748,337 did not antagonize the positive inotropic effect of CGP12177, but the non-specific β-AR antagonist bupranolol (1 µM) did. Altogether, these results suggest that under physiological conditions (37 °C), β3-AR may not induce contractile effects on atrial myocytes and caution against potential off-target effects of some β3-AR agonists on β1/β2-AR, especially at high concentrations. In line with this, Mo et al. [30] observed an increase in contractility of human atrial trabeculae incubated with the β3-AR agonist, mirabegron, but only at concentrations from 1 to 10 µM of mirabegron—well over the 1 µM threshold for agonist specificity—but not at 0.1 µM. The effect was abrogated upon co-incubation with the β1-AR antagonist, CGP20712A (300 nM), but not by the β3-AR antagonist, L-748,337 (100 nM). Not surprisingly, this inotropic effect of 10 µM mirabegron was attributed to off-target stimulation of β1-AR. Therefore, a careful analysis of experimental models and conditions used may help to resolve the apparent discrepancies between the observations reported above. Most would point to an effect of β3-AR opposite to that of β1/β2-AR on human ventricular muscle, while effects on atrial contractility remain to be further investigated. Future studies should carefully balance the relative concentrations of β3-AR agonists with saturating concentrations of β1/β2-AR antagonist [18] to firmly establish a β3-AR effect in human tissues.

### 3.3. β3-AR Signaling

The β3-AR/cGMP downstream signaling involves not only the eNOS pathway, but also nNOS, with the two enzymes cooperating to maintain signaling in the face of catecholaminergic stress [31]. In this latter study, the authors observed that decreases in sarcomere shortening and Ca^2+^ transient induced by β3-AR stimulation are abolished in cardiac myocytes isolated from knockout mice lacking either eNOS or nNOS (eNOS^−/−^ and nNOS^−/−^, respectively); the same was observed in cardiac myocytes treated with a specific nNOS inhibitor (SMTC). This regulation of the β3-AR response was explained by a nNOS-eNOS crosstalk in which nNOS is required to inhibit xanthine oxidoreductase (XOR)-dependent superoxide anion production, thereby protecting eNOS function from oxidative uncoupling.

The β3-AR-mediated attenuation of contractility and positive lusitropic effects can be attributed to the well-described NOS/NO/cGMP/PKG signaling regulation of Ca^2+^ handling and sarcomere function in cardiac myocytes (reviewed in [32]). β3-AR regulation of the Ca^2+^ transient may operate both through the NOS-dependent inhibition of L-type Ca^2+^ channels (LTCC)-mediated Ica current [16] (thereby attenuating EC coupling) and through improved Ca^2+^ re-uptake during cardiac myocyte relaxation through PKG-mediated phosphorylation of phospholamban. cGMP/PKG signaling downstream β3-AR may also regulate cardiac diastole by decreasing myofilament Ca^2+^ sensitivity and modulating myocardial stiffness through troponin I (TnI) [33] and titin [34] phosphorylations (Figure 1A). Additionally, the cGMP-to-cAMP crosstalk can modulate myocyte contractility through subcellular pools of phosphodiesterase isoforms, particularly isoforms 2 and 3 (PDE2 and PDE3, respectively). In neonatal rat cardiac myocytes expressing a FRET cAMP sensor, selective PDE isoform inhibition revealed that the NOS-dependent anti-β1/β2AR inotropic effect of β3-AR stimulation involves a cGMP-mediated inhibition of cAMP through PDE2 activation [35]. This result was recently confirmed in healthy adult rat cardiac myocytes expressing the cAMP biosensor Epac1-camps in which β3-AR stimulation led to a reduction of approximately 10% of cAMP production obtained with forskolin (direct AC stimulation independent of β1/β2AR stimulation). This effect of β3-AR stimulation on cAMP levels was abolished in the presence of a PDE2 inhibitor [23]. PDE2 (a *cGMP-*activated *PDE*) and PDE3 (a *cGMP-*inhibited *PDE*) are able to hydrolyze both cAMP and cGMP, but with higher affinity and/or Vmax activity for cAMP, while PDE5 is a selective cGMP hydrolase. Interestingly, in cardiac myocytes expressing a FRET-cGMP biosensor, the authors also observed that cGMP production induced by β3-AR stimulation is itself under the control of PDE5 and PDE2.

Other targets of NOS/sCG may participate in β3-AR regulation of myocyte contractility. In ventricular myocytes from sheep with HF, activation of β3-AR improved contractility by increasing Na^+^/K^+^ pump activity, through the prevention of oxidative alteration of the β1 subunit of the pump. This beneficial effect was abolished by NOS or sGC inhibition (with L-NAME or ODQ, respectively) [36]. In guinea-pig ventricular myocytes, β3-AR stimulation was also shown to modulate cardiac potassium channel function by decreasing the slow component of the delayed rectifier potassium current (I_Ks_), but the functional implications on contractility (or rhythm control) remain to be investigated [37] (Figure 1A).

Finally, in addition to the fine-tuning of myocyte excitation–contraction coupling, β3-AR modulates cardiac function by directly regulating human coronary arteries relaxation [3], but also vascular systemic endothelial function [2,20,38], mainly—but not exclusively—through NOS/NO dependent mechanisms. Paracrine effects of β3-AR signaling from endothelial cells, but also to cardiac fibroblast [26] and from perivascular/epicardial adipose tissues are emerging as targets of interest for cardiac regulation and remodeling (see Section 4 and Section 5).

## 4. β3-AR Expression and Function in the Diseased Heart

As mentioned above, β3-AR expression is increased in human failing hearts [15], but also in animal models of HF [16,17], diabetic hearts [39] and sepsis [40,41]. Conversely, in HF, β1-AR are downregulated by almost 50%, leading to an unbalanced β1/β2-AR expression ratio from 80:20 in physiological condition to 60:40 in pathology, while β2-AR abundance remains stable but the receptor is functionally desensitized [21,42,43]. Interestingly, the recent study of Schobesberger et al. [23] combining FRET and SICM techniques shows that in failing rat cardiomyocytes, β3-AR partially translocate from T-tubules to the membrane crest, which is associated with a two-fold reduction in FRET-cGMP signal in response to the saturating concentration of isoproterenol. Such functional impairment could also be explained by a partial disruption of sGC co-localization with caveolin-3 in failing myocytes. Consistently, β3-AR-dependent cGMP production was impaired as well as the cGMP/cAMP cross-talk with impaired β3-AR-induced decrease in cAMP despite PDE2 predominant activity. These observations argue for a pathological spatial rearrangement of the β3-AR/sGC/PDE2 signalosome in severe HF, with impaired functional antagonism of the β3-AR/cGMP against β1-AR/cAMP that may contribute to further deterioration of cardiac remodeling and function. Note, however, that failing rat cardiac myocytes in this study were obtained 16 weeks after myocardial infarction, with a severe HF phenotype known to cause T-tubule disruption [44] and PDE2 overexpression [45]. These features may not be entirely reproduced in a less severe phenotype, such as early cardiac structural disease without severe reduction in systolic function.

As a brief summary of the chronological evolution of HF with reduced ejection fraction (HFrEF), during a first adaptive period, sustained catecholamine (adrenaline, noradrenaline) stimulation is recruited, and required, to maintain cardiac contractile function for appropriate blood supply to peripheral organs (compensated stage). However, chronic (over)stimulation of the receptors progressively leads to β1/β2AR desensitization and subsequent loss of contractile function (decompensated stage), in an interdependent manner with morphological hypertrophic-to-dilated cardiac remodeling and fibrosis [46,47]. In the context of this progressive evolution, any influence to acutely attenuate positive inotropic signaling could be deleterious for cardiac function preservation in the short term, while a similar influence in the long term could prevent and/or resolve functional and morphological remodeling. This dual-sided paradigm would largely explain the discrepancies reported on the protective or deleterious effects of β3-AR stimulation in the diseased heart, depending on the phenotypic stage and/or HF model (myocardial infarction, hemodynamic pressure overload, ischemia-reperfusion, etc.), disease severity and timing in the progression of pathological remodeling.

Accordingly, in a dog model of rapid pacing-induced HF associated with an increase in cardiac β3-AR expression, acute β3-AR stimulation further decreased contractility and Ca^2+^ transient of isolated ventricular myocytes from failed hearts [16]. A similar negative inotropic effect of β3-AR stimulation was reproduced in vivo upon i.v. infusion of BRL37344 in dogs with HF [48], leading to conclude that β3-AR upregulation may contribute to progression of the cardiac dysfunction. Note that in this model, acute effects of β3-AR stimulation were observed at the early stage of a mild HF and that systemic hemodynamic effects of BRL37344 perfusion were not taken into consideration. An increase in β3-AR expression was also observed in human myocardium from septic patients [40]. A similar observation was reported in a sepsis model of HF in mice, in which in vivo treatment with a β3-AR agonist (CL316243) exacerbated cardiac dysfunction, while a β3-AR antagonist (SR59230A i.p. injections) prevented cardiac dysfunction in parallel with decreased iNOS expression and left ventricular NO concentration [41]. However, direct effects of β3-AR agonist/antagonists administered systemically on cardiac iNOS should be interpreted with caution. An increase in iNOS-related oxidative stress associated with chronic β3-AR stimulation has also been reported to exacerbate atrial fibrillation and remodeling in a dog model of atrial pacing, while β3-AR antagonist prevented atrial dysfunction [49]. β3-AR abundance was also reported to be upregulated in human diabetic hearts [39], and could contribute to altered cardiac inotropic response in diabetic cardiomyopathy [50].

In contrast to the above, the majority of studies report cardioprotective effects of β3-AR stimulation on heart function and remodeling, which mainly involve (i) preservation of contractile function mediated by NOS-signaling and antioxidant effects, (ii) antihypertrophic and antifibrotic effects and (iii) metabolic effects. As β3-AR signaling regulates cardiac relaxation—notably through PKG-downstream signaling—and prevents hypertrophic remodeling, it recently appeared as a promising therapeutic target for HF with preserved ejection fraction (HFpEF). These points are discussed in the following section.

## 5. Cardioprotective β3-AR Signaling on Heart Function and Remodeling

### 5.1. Preservation of Contractile Function by β3-AR: NOS Signaling and Antioxidant Effects

Cardioprotective effects of β3-AR signaling against HF were initially inferred from the phenotype of knockout mice lacking the receptor [51]. β3-AR^−/−^ mice submitted to pressure overload-induced HF (by transverse aortic constriction—TAC) displayed exacerbated left ventricular (LV) contractile dysfunction (reduced EF) and cardiac remodeling compared to wild type (WT) mice. These deleterious effects of β3-AR genetic deletion were associated with decreased eNOS phosphorylation on its activation site (Ser^1177^) and increased uncoupling, responsible for higher NOS-dependent superoxide production. On the contrary, β3-AR stimulation (BRL37344; 0.1 mg/kg/h by osmotic minipump) conferred cardioprotection against haemodynamic stress [52]. In the latter study, β3-AR stimulation prevented the loss of NO production and reduced superoxide generation induced by TAC via a nNOS-dependent mechanism. In a different model of myocardial infarction (by left coronary artery ligation), systemic infusion of BRL37344 preserved cardiac contractile function with reduced fibrosis and apoptosis [53]. Further dissecting nNOS-mediated beneficial effects in neonatal rat cardiomyocytes stimulated with endothelin-1 or norepinephrine to produce hypertrophy, β3-AR protection from cell growth and oxidative stress was explained by increased phosphorylation at Ser^1412^ and mostly dephosphorylation at Ser^847^, associated with increased nNOS activity, higher cGMP synthesis and decrease in ROS production. Such protective effects were lost in mouse cells expressing phosphomimetic nNOS mutants on Ser^847^. Moreover, inhibition of Gαi protein (PTX incubation) blocked BRL37344-induced nNOS post-translational modifications and ROS reduction. Similar cardiac nNOS post-translational modifications were reproduced upon BRL37344 treatment of mice submitted to 3 weeks of TAC [54]. Interestingly, beneficial effects of β3-AR stimulation on HF were also reported to be part of the cardioprotective effects of a commonly used β-AR blocker. Indeed, in a mitral valve regurgitation-induced heart failure model in dogs, metoprolol (β1-AR antagonist) administration prevented oxidation of sGC and promoted β3-AR/sGC-NO-cGMP coupling in specific membrane microdomains, but away from caveolae. This activation of β3-AR/cGMP pathway was again attributed to nNOS activation by increased phosphorylation at Ser^1412^ [17]. Such improvement of β3-AR/sGC-NO-cGMP coupling with the commonly used metroprolol may, in part, explain the widely observed therapeutic benefit of β1-AR blockade in HF. Given the cooperative effects of eNOS and nNOS on cardiomyocyte contractility in cardiac diseases (reviewed in Farah et al. [32]), and the previous observation that nNOS translocates from the sarcoplasmic reticulum to the plasma membrane in failing human hearts [55], the antioxidant effect mediated by the nNOS-eNOS crosstalk and subsequently preserved NOS/sGC/cGMP signaling would reinforce the cardioprotective effect of β3-AR stimulation in the failing myocardium.

In addition, preservation of the Na^+^/K^+^ pump function upon β3-AR stimulation also contributes to the attenuation of congestive cardiac remodeling induced by coronary ligation in rabbits [56]—a phenomenon similarly observed in a diabetic heart model in which β3-AR stimulation prevents inactivation of the Na^+^/K^+^ pump induced by NADPH oxidase-dependent oxidation of its β1-subunit [57]. Preservation of Na^+^/K^+^ pump function would oppose the Na^+^ overload commonly observed in failing myocytes and restore EC coupling and systolic function (Figure 1B).

β3-AR activation of eNOS and nNOS was also involved in preventive cardioprotective effects of exercise training against myocardial infarction [58], as well as in the protection against cardiac ischemia-reperfusion (IR). Administration of β3-AR agonists at reperfusion increases eNOS phosphorylation at Ser^1177^ and nNOS expression, and decreased infarct size observed 24 h after IR; this protection was lost in eNOS^−/−^ and nNOS^−/−^ mice [59]. Furthermore, pre-perfusion of a β3-AR agonist before IR preserved long-time LV contractile function and reduced infarct size in rodents and large pigs, which may involve NOS-dependent inhibition of mitochondrial permeability transition pore (mPTP) opening—a trigger of apoptosis and massive oxidative stress—during reperfusion [60]. Accordingly, administration of the β3-AR agonist (BRL37344, 1 μM) pre, per IR or at early reperfusion reduced the subsequent infarct size in isolated rat hearts [61]. However, this beneficial effect could not be reproduced with mirabegron, an agonist with high specificity for the human β3-AR in a swine model of IR in vivo; upon administration before reperfusion, the drug had no effect on LV function recovery nor on infarct size after 7 or 45 days post-IR [62]. These discrepancies are most likely explained by differences in experimental models and species and point to the necessity to take into account the specificities of β3-AR pharmacology for potential therapeutic applications.

In recent years, β3-AR/PKG signaling emerged as a promising therapeutic target in heart failure with preserved ejection fraction (HFpEF). Diastolic dysfunction in HFpEF patients mainly results from the combination of increased cardiomyocyte stiffness with LV hypertrophic remodeling and interstitial fibrosis [63,64,65]. Cardiomyocyte stiffness results from both increased myofilaments Ca^2+^ sensitivity and higher titin stiffness, related to reduced PKG activity in the myocardium of HFpEF patients [66] and subsequent lower phosphorylation of these targets [67,68,69]. Therefore, β3-AR stimulation, by improving NOS/PKG signaling, should restore the phosphorylation of sarcomeric proteins (Tni, MyPBC, titin) but also improve the regulation of Ca^2+^ handling for cardiac myocytes relaxation. Moreover, beneficial effects of β3-AR signaling on hypertrophic remodeling and fibrosis may reinforce its therapeutic potential in both HFpEF and HFrEF.

### 5.2. Antihypertrophic and Antifibrotic Effects of β3-AR Signaling

Our group and others showed that activation of cardiac myocyte β3-AR protects against the deleterious effects of chronic adrenergic stimulation, particularly against hypertrophic remodeling and myocardial fibrosis.

An antihypertrophic effect of β3-AR was initially observed by pharmacological stimulation of primary myocytes and in vivo infusion of β3-AR agonists in rodents [52,54]. The direct involvement of cardiac β3-AR was demonstrated using the mouse model harboring a cardiac-specific expression of the human β3-AR (β3-Tg) (see above) where the receptor is expressed at a level similar to that observed in the human heart. Moderate expression of the human β3-AR protected against remodeling induced by catecholaminergic (isoproterenol injections) or hemodynamic (TAC) stress, while the protection was lost in another mouse model of inducible (Cre-Lox) deletion of β3-AR [22,26].

Strikingly, the β3-AR-mediated protection extended to myocardial interstitial fibrosis in the above models. A similar antifibrotic effect was also observed following myocardial infarction upon treatment of mice with β3-AR agonists [53] along with a reduced scar area and apoptosis [53,59] yet was not replicated in mice submitted to TAC and treated with the β3-AR agonist BRL-37344; this was most likely explained by the low basal level of expression of endogenous cardiac β3-AR in mice and the short timing of investigation following TAC (i.e., one and three weeks post-TAC vs. 9 weeks in all other studies) [52].

As extensively described in the previous section, β3-AR signaling in cardiac myocytes is linked to the PKG/cGMP/NOS pathway. The cardioprotective effects of β3-AR against hypertrophic remodeling and fibrosis following neurohormonal and haemodynamic stress were also shown to be mediated by nNOS [22,26,52,54] and to implicate a decrease in ROS production [26,31,52,54]; as mentioned in previous sections, this effect is attributed to protective nNOS-mediated inhibition of XOR [31,70] which in turn prevents eNOS oxidative uncoupling [31,51] (Figure 1B).

**Figure 2:**
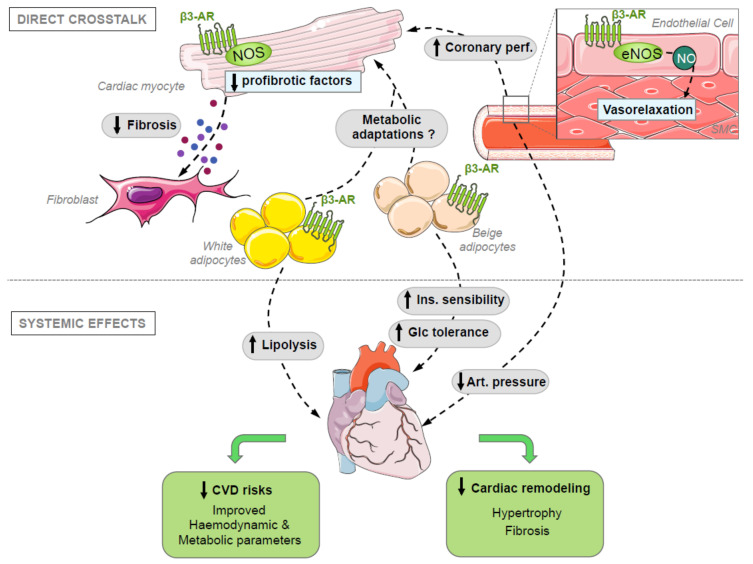
B3AR-mediated cardiovascular protection through paracrine and systemic signaling. β3-AR are expressed in human white and beige adipocytes, as well as coronary (and peripheral) endothelial cells and cardiac myocytes. In the latter, NOS-mediated signaling decreases oxidant stress (see Figure 1B), which results in a decrease in the synthesis of myocyte-derived profibrotic factors (e.g., CTGF, TGFB2) acting on neighboring cardiac fibroblasts. NO-dependent vasorelaxation contributes to the regulation of coronary perfusion and, possibly, systemic blood pressure (although this probably integrates additional influences—e.g., on kidney function). β3-AR-mediated beiging of (human) visceral adipocytes and lipolysis in white adipocytes contributes to improvements in peripheral metabolism, with improved insulin sensitivity and glucose tolerance. Additional (still undiscovered) metabolites or adipokines may exert direct influences on cardiac metabolism and/or remodeling. Ultimately, these pleiotropic effects would decrease cardiovascular risk factors and attenuate myocardial adverse remodeling.

Mechanistically, the reduced ROS levels following β3-AR activation attenuate fibrosis through reduced release of paracrine profibrotic agents in β3-AR expressing myocytes; in superfusion experiments using conditioned media and secretome analysis, connective tissue growth factor (CTGF) was identified as one of the major contributors, as its silencing in cardiac myocytes significantly attenuated the pro-fibrotic effect upon their stimulation with phenylephrine [26] (Figure 2). In addition to the reduction in ROS production through the PKG/cGMP/nNOS pathway, our group recently demonstrated that the blunted hypertrophic remodeling in β3-Tg was in part attributable to the sustained activation of AMP activated protein kinase (AMPK) [71], resulting in enhanced autophagic flux. Of interest, AMPK is a bona fide nNOS kinase (on Ser^1412^) [72] and eNOS-activating kinase in cardiac muscle, particularly upon exercise training in mice [73]. However, additional downstream effects of AMPK are also more in line with its classical role as a metabolic regulator, as detailed below.

### 5.3. Role of Metabolism in β3-AR-Mediated Cardioprotection

AMPK was found to colocalize with β3-AR at the caveolae. Transgenic human β3-AR expression sustained the activation of AMPK and its downstream effectors in the face of neurohormonal and haemodynamic stress. Conversely, silencing of crucial AMPK α1/α2 catalytic subunits in cardiac myocytes significantly blunted the antihypertrophic effect of β3-AR [71]. While AMPK antihypertrophic effect has been linked with an increase in autophagic flux in the stressed heart as mentioned above, AMPK cardioprotective effect may well implicate additional roles on substrate availability and energy production given the upstream regulator role of AMPK in cellular energy, oxidation and mitochondrial biogenesis [74]. Indeed, in the face of stress, AMPK prevents adverse remodeling by limiting protein synthesis, fibrosis and proliferation of myofibroblasts while increasing the autophagic flux as well as restoring the cellular energy level [74,75]. This last process combines favoring ATP-producing processes (glucose utilization and fatty acid oxidation) together with the stimulation of substrate availability and mitochondrial biogenesis [76]. Given the importance of metabolic changes in the progression of HF, the cardioprotective effect of AMPK activation mediated by β3-AR may well involve these additional roles on cellular bioenergetics.

β3-AR have traditionally been linked to metabolic effects in adipose tissue—e.g., through activation of brown fat in rodents and beiging of white adipocytes in rodents and humans. The transcription factor, Prdm16, is a main regulator of the beiging process in white adipose tissue (WAT) [77,78]. In adipocytes, β3-AR stimulation upregulates Prdm16 expression [79], while conversely, Prdm16/ebpβ complex increases β3-AR transcription by transactivation of the β3-AR promoter [80]. A recent study showed that Prdm16 in cardiac myocytes is required to inhibit hypertrophic remodeling and fibrosis [81]. Prdm16 deficiency was associated with apparent mitochondrial defects and changes in transcriptional pattern suggesting a switch from fatty acid oxidation towards glucose utilization. In cardiac myocytes, the relationship between Prdm16 and β3-AR has not yet been investigated but based on these latest observations could reveal new mechanisms by which β3-AR protects the heart against adverse remodeling as well as additional implications of B3-AR in metabolic flexibility.

In addition to oxidative stress handling, β3-AR was strongly associated with metabolism in a proteomic study analyzing differentially expressed proteins from hearts of β3-AR systemic KO mice compared to WT [82]. This study highlighted a significant link with lipid metabolism and transport. Moreover, differentially acetylated proteins in the β3-AR KO hearts suggested an impact on the TCA cycle, further implicating β3-AR in cardiac metabolic pathways. Nevertheless, the study was performed in a systemic KO mouse model where contribution deriving from β3-AR deficiency in adipose tissue cannot be ruled out [82].

## 6. Systemic Stimulation of β3-AR

Similarly, the interpretation of cardiac effects of β3-AR agonists administered systemically must take into account potential effects mediated by β3-AR in adipose tissue. The latter deserve special consideration, as improvements in the overall metabolic status—i.e., decreased triglyceridemia, and decreased obesity, with increased insulin sensitivity and/or β3-AR-mediated endocrine/paracrine signaling from adipose tissue—can greatly affect the cardiovascular system.

In brown adipose tissue, non-shivering thermogenesis in rodents has long been shown to be regulated by the activation of the β3-AR coupled to Gαs leading to activation of AC and intracellular of cAMP/PKA signaling [83]; this leads to enhanced lipolysis via activation of ATGL and other lipases, increased mitochondrial biogenesis and the activation and upregulation of the uncoupling protein 1 (UCP1) responsible for the mitochondrial uncoupling [84]. As a metabolic waste for excessively accumulated lipids, activation of thermogenesis in brown adipose tissue with β3-AR agonists has raised initial hopes as a treatment for obesity and metabolic syndrome. Despite promising results on rodent models of obesity and metabolic syndrome showing a reduction in obesity, weight loss and improvement in glucose homeostasis with Β3-AR agonists [85,86], clinical trials testing the same in humans performed poorly, at best [87,88,89]. The disappointing results were originally attributed to the poor selectivity of agonists for human β3-AR causing weak stimulation of BAT and off-target beta-adrenergic cardiovascular side-effects. However, the development of the new selective β3-AR agonist, mirabegron, and its approval by health authorities for the treatment of overactive bladder disease [90] led to new pilot clinical trials assessing BAT stimulation following acute or more chronic treatment with this drug. The results showed variable BAT stimulation with the low/clinically recommended dose (50 mg/day) of mirabegron [91,92], while higher doses (100 mg/day and above) consistently stimulated BAT and increased resting energy expenditure [93,94]. The variable response to low doses was first attributed to differences in subject populations (lean vs. obese subjects) as BAT is reduced in obese and elderly subjects [95]. However, very recent work challenging the role of β3-AR in human brown adipose tissue attributes the thermogenic effect to another beta-adrenergic receptor [96,97], namely to β3-AR. According to these studies, the β3-AR is very weakly expressed in non-immortalized human brown adipocytes and unresponsive to mirabegron; additionally, unlike β3-AR, silencing of the β3-AR had no impact on the respiration level under agonist stimulation. This new paradigm would explain the inability to activate human BAT with the clinically-used dose of mirabegron (50 mg/day), while BAT activation at higher doses (200 mg/day) would result from off-target effects on other beta-adrenergic receptors generally accompanied by secondary cardiovascular effects on heart rate and systolic blood pressure [97]. Of note, the β3-AR is upregulated under silencing of β2-AR and may well partly participate in compensating mechanisms of thermogenesis—e.g., when highly desensitizable β2-AR may become inoperant. The variability in the efficacy of low-dose mirabegron to stimulate brown adipocytes may also result from interindividual differences not only in BAT volume but also in β3-AR expression in BAT [91,97,98].

While the argument for β2-AR mediated thermogenesis in human BAT is solid, it probably does not extend to human beige adipose tissue where the evidence points to a β3-AR mediated signaling. Beige adipocytes with thermogenic ability interspersed within white adipose tissue (WAT) were recently identified in mouse and human [99]; in addition, the beiging process of white adipocytes is mediated by β3-AR stimulation in rodent models and involves the transcription factor Prdm16 [78,100] while this beiging capacity is kept under control by Foxp1-mediated repression of β3-AR transcription [80]. Interestingly, while originally thought to follow a unique thermogenesis process similar to BAT, recent data demonstrate that beige adipocytes are able to perform not only UCP1-dependent but also UCP1-independent thermogenesis, both processes being governed by AC downstream signaling. Although the exact signaling pathway remains unclear, the non-canonical UCP1-independent pathway implicates heat production from the ATP-dependent Ca^2+^ cycling by the sarco/endoplasmic reticulum Ca^2+^ ATPase 2b (SERCA2b) when Ca^2+^ transport is uncoupled from ATP hydrolysis [101]. In beige adipocytes, this process is accounted for 70% by β3-AR activation and for 30% by α1-AR activation in mouse models. A similar thermogenic process has been previously described in skeletal muscle, but unrelated to β3-AR signaling as the receptor is not expressed in this tissue [102]. Whether a similar, β3-AR dependent process operates in cardiac myocytes has never been investigated. Most importantly, in rodent beige adipocytes, while UCP1 dependent thermogenesis relies primarily on fatty acid use, SERCA2b-dependent thermogenesis correlates with an intensified glucose use supported by increased glucose uptake, glycolysis and glucose oxidation. This new thermogenic process acting as a “glucose sink” underlies the observation that beige adipocytes regulate glucose tolerance and insulin sensitivity [101,103]. In humans, a 10 day-treatment with 50 mg/day of mirabegron induced a beiging of white adipose tissue significantly higher than that induced by cold exposure in lean and obese subjects [92,98]. Human white adipocytes do express β3-AR where its stimulation triggers lipolysis (reviewed in [104]. While a single low dose of mirabegron may not produce measurable lipolysis in human WAT [97], recent studies clearly showed a significant lipolysis following chronic administration of the drug [92,94,98]. Aside from the low number of patients included in these pilot studies, the discrepancy between these results may well be explained by the different timing of administration of the agonist—i.e., chronic treatment with mirabegron may be needed to reach a level of lipolysis that is detectable in the plasma, also bearing in mind that β3-AR expression is upregulated by chronic adrenergic stimulation in some tissues.

At this stage, experiments performed with systemic β3-AR stimulation do not allow to discriminate beneficial effects linked to WAT lipolysis from those due to beige adipocytes stimulation and thermogenesis. Nevertheless, stimulation of adipocytes by mirabegron was associated with a significant improvement in glucose tolerance and insulin sensitivity in both healthy and obese subjects [92,94] despite no measurable change in fasting glucose and insulin (Figure 2). Although not placebo-controlled, the study by Finlin et al. in obese subjects ended with 5 of 9 patients no longer prediabetic by the end of the study according to the American Diabetes Association criteria. Therefore, by reducing cardiovascular risk factors, a long-term treatment with mirabegron is likely to reduce the incidence of cardiovascular diseases through a systemic effect, although this remains to be tested prospectively in a proper RCT. These observations also highlight a crosstalk between adipocytes and remote organs such as, possibly, beta cells in the pancreas, among others. Of particular interest is the finding that beiging and lipolysis of subcutaneous adipose tissue following mirabegron treatment causes a significant switch of the skeletal muscle type towards a higher composition of oxidative type I fibers paralleled with an increase in PGC1α and its downstream effectors (such as TFAM1, COXIV and PLIN5); as skeletal muscles do not express β3-AR, this effect was fully attributed to activation of β3-AR in adipocytes and a subsequent paracrine effect. Indeed, it could be recapitulated in vitro by culturing human myotubes with conditioned media of human adipocytes treated with β3-AR agonists [92]. However, the factors responsible for these remote effects, whether metabolites or adipokines remain to be identified.

In a manner similar to this observation in skeletal muscle, a single β3-AR agonist injection in a WT mouse was found to strongly upregulate Perlipin 2 (PLIN2) and Perlipin 5 (PLIN5) expression in cardiac myocytes [105]. Again, this effect was attributed to remote signals from adipocytes given the low, basal endogenous levels of β3-AR in healthy mouse cardiac myocytes. Such remote effect from adipocytes triggered an elevation in cardiac levels of triacylglycerides (TAG), an increase in size and number of cardiac lipid droplets (LD), an upregulation of PLIN5 localization at the LD-mitochondria interface as well as an increase in cristae in mitochondria not associated to LD. The precise adipocyte-derived element responsible for these changes is not yet known; whether this is due to elevation of plasma TAG, non-esterified fatty acid (NEFA) or rather unidentified adipokine(s) remains to be examined. The downstream effect on metabolism is also unclear, but these morphological observations could point towards an increased beta-oxidation in mitochondria not associated with LD, while the LD associated with less active mitochondria would favor lipid esterification altogether reducing cellular NEFA levels and lipotoxicity. To date, clinical studies have not thoroughly investigated patients with metabolic syndrome at risk of, or already developing, HF and the effect of a chronic treatment with a β3-AR agonist on cardiac function in these patients. In addition to direct effects on β3-AR expressed in human cardiac myocytes, systemic effect from adipocytes may hypothetically influence cardiac myocyte metabolism through adjustments of substrates availability, as well as increased mitochondrial biogenesis and fatty acid oxidation, in correlation with increased levels of PGC1aα similar to what is observed in skeletal muscle [92].

Systemic administration of β3-AR agonists is likely to affect the adipose tissue in different locations—i.e., visceral, epicardial or perivascular—with various effects depending on their content in beige adipocytes and lipolytic capacity. From the perspective of clinical applications, the resulting cross-talk between adipocytes and cardiac myocytes is of particular importance as recent studies highlight a higher content of beige adipocytes in human visceral (VAT) than in subcutaneous adipose tissue [106] contrary to rodent models [107]. In addition, a recent study using single cell RNA sequencing demonstrated that β3-AR stimulation triggers a strong induction of adipocyte stem cells differentiating into beige adipocytes in VAT [108]. Any biological change in VAT is likely to have an impact on human health, since VAT correlates with an elevated risk for diabetes, hypertension and atherosclerosis [109,110]; VAT was recently associated with cardiac aging through its secretion of profibrotic factors such as osteopontin that stimulates myocardial fibroblasts and decreases fibroblasts senescence [111].

Apart from VAT, stimulation by β3-AR of the perivascular adipose tissue (PVAT) was shown to generate a cAMP-dependent release of NO that attenuates the contractile response of rodent mesenteric arteries [112]. In another recent study, β3-AR agonist stimulation of human epicardial adipose tissue (EAT) ex vivo triggered spontaneous contractions of the underlying atrial myocardium [113], an effect reproduced in superfusion experiments. While this observation would suggest EAT-induced susceptibility for atrial arrhythmias, none of the numerous pre-clinical and clinical trials using systemic β3-AR stimulation reported any induction of atrial arrhythmias, even at the high dosage of 200 mg of mirabegron [114]. Altogether, these data highlight the importance to investigate β3-AR effects in the different adipose depots in vivo and their implication on the heart in healthy and pathological conditions.

## 7. β3-AR Agonists and Clinical Trials

Based on the evidence reviewed in the previous sections, β3-AR represent a promising therapeutic target for the treatment or prevention of cardiovascular diseases (CVD), be it through their systemic or direct activation in cardiac myocytes, in particular given their increased expression in the pathological myocardium [15]. This can now be tested clinically by “re-purposing” the specific β3-AR agonist, mirabegron that was developed and approved in Europe, USA and Japan for the treatment of overactive bladder disease, where the drug improves bladder filling by activating β3-AR in the detrusor muscle leading to myorelaxant effects [90].

Evaluation of the cardiovascular safety of mirabegron is an important prerequisite given the recent report of mirabegron-mediated increase in human atrial force, albeit at high concentration in vitro (see discussion above). This side-effect of mirabegron was attributed to an accrued norepinephrine release from sympathetic nerves leading to stimulation of the β1-AR response in isolated atrial trabeculae [30]. Regardless, cardiovascular safety has been thoroughly evaluated in numerous Phase III clinical trials of mirabegron in Overactive Bladder Disease and did not raise major concerns. These trials revealed an increase in heart rate (HR) of around 1 bpm and in systolic blood pressure (SBP) of less than 1 mmHg only. With the current recommended dose of 50 mg/day, the difference in HR and SBP was not clinically relevant, nor was it associated with cardiovascular adverse events such as tachycardia or palpitations. The QTc interval was only found to be prolonged at the supratherapeutic dosage of 200 mg [114]. Therefore, the availability of this new drug offers the possibility to test the potential beneficial effect of activation of β3-AR to prevent and/or delay myocardial remodeling in patients at high risk of developing HF as add-on therapy.

The first randomized trial on CVD examined the effect of mirabegron on 70 patients suffering from HFrEF. The rationale for this trial was based on preclinical data in failing sheep in vivo and rabbit cardiac myocytes where β3-AR stimulation was found to reduce the detrimental increase in intracellular Na^+^ levels—the effect involved protection of the Na^+^-K^+^ -ATPase activity from oxidative inactivation, as developed in Section 2 [36]. In the BEAT-HF trial (NCT01876433), patients with NYHA class II-III HF and LV dysfunction (LVEF ≤ 40%) received up to 300 mg mirabegron daily and were monitored over 6 months. The primary endpoint (increase in the LV ejection fraction) was not reached [115], but exploratory analysis of these results revealed that in the subgroup of subjects having more severe LV dysfunction at baseline (LVEF < 40%), the active treatment led to a significant improvement of ejection fraction. A follow-up trial (BEAT HF II) (NCT03926754) is currently taking place to investigate the same dose of 300 mg/day in patients selected for NYHA class III-IV HF with a more severely reduced ejection fraction (LVEF < 35%). Note that in both trials, patients are treated with full-dose β1/β2-AR blockers to prevent off-target side-effects.

While the BEAT HF trials test the beneficial effects of a chronic β3-AR activation in advanced HF, no trial has yet tested the reverse hypothesis—i.e., that a β3-AR antagonist, administered acutely, may improve LV function by antagonizing the negative inotropic effect of β3-AR at the early phase of acute HF (see Section 3 above). The recent development of very specific antagonists at human β3-AR may soon fill this gap.

In a porcine model of pulmonary hypertension, β3-AR agonists also improved right ventricular (RV) performance and pulmonary hemodynamics, particularly pulmonary vascular resistance (PVR) [116]. Accordingly, the beneficial effect of mirabegron is now being tested in a phase II randomized double-blind trial (SPHERE-HF) (NCT02775539) assessing the effect of mirabegron on PVR on 80 patients suffering from chronic pulmonary hypertension secondary to HF who will receive placebo or 50 to 200 mg/day of mirabegron [117]; the inclusion phase was expected to end by June 2020.

As mentioned in Section 4, β3-AR emerged in recent years as a promising therapeutic target in HFpEF. Indeed, β3-AR stimulation decreases LV hypertrophic remodeling and interstitial fibrosis in preclinical models [22,26]; it would also be expected to decrease myofilaments Ca^2+^ sensitivity and lower titin stiffness; and it mediates NOS-dependent vasorelaxation in the human coronary microvasculature [3]. β3-AR agonists would then preserve myocardial perfusion, prevent cardiac remodeling and improve diastolic LV relaxation in patients with structural heart disease (stage B, AHA) at high risk of development or worsening of HFpEF [118]. This is the rationale for the current phase IIb clinical trial, BETA3_LVH (NCT02599480). Accordingly, this multi-centric placebo-controlled randomized trial included 296 patients assigned to receive placebo or 50 mg/day of mirabegron over a period of 12 months. The primary endpoints assess changes in LV mass index (by cardiac MRI) and in LV diastolic function (E/E’), while secondary endpoints include the effect on cardiac fibrosis, left atrial volume index, exercise capacity as well as specific biomarkers reflective of myocardial remodeling. In a transdisciplinary endeavor, additional parameters will be monitored to evaluate the systemic effect of mirabegron on endothelial function measured by both pulse amplitude tonometry and NO bioavailability in erythrocytes by EPR spectroscopy [119]; a sub-study specifically measures the effect of mirabegron on adipose tissue activity by18FDG-PET combined with CT scan of BAT and several metabolic parameters. The trial is expected to be completed by the end of 2021 [120].

## 8. Conclusions

The substantial body of evidence reviewed above clearly indicates pleiotropic roles of the human β3-AR beyond metabolic regulation in adipose tissue. Direct and systemic, indirect influences on the contractility, remodeling and, possibly metabolism of the cardiac muscle justify past and current efforts to harness the human β3-AR for the treatment of specific forms of HF. Careful determination of the dose and timing of administration of the newly available agonists (and perhaps, antagonists) at the human β3-AR should enable the accumulated knowledge to be translated into new avenues of treatment for this deadly disease.
